# Evaluation of hand‐arm vibration (HAV) exposure levels among grounds maintenance workers: An observational human exposure measurement study

**DOI:** 10.1002/hsr2.731

**Published:** 2022-07-20

**Authors:** Jonghwa Oh

**Affiliations:** ^1^ Department of Environmental Health Sciences University of Alabama at Birmingham Birmingham Alabama USA

**Keywords:** backpack blower, daily vibration exposure, grass trimmer, hand‐arm vibration (HAV)

## 1 INTRODUCTION

Long‐term, excessive exposure to hand‐arm vibration (HAV) through vibrating hand tools can induce hand‐arm vibration syndrome (HAVS) which is a complex of peripheral vascular, neurological, and musculoskeletal disorders.^1^ The development of HAVS depends on a variety of factors such as vibration magnitude, frequency, direction, exposure duration, posture, and contact force.^1^ However, there are no standardized methods for HAV exposure assessment. Many studies traditionally have measured acceleration values directly from tools by mounting an accelerometer on the tool handle^2^, ^3^, ^4^, ^5^, ^6^ while more recent studies have measured vibration magnitude at the hand‐handle interface using hand‐held/hand‐mounted adapters in which an accelerometer is attached to or inserted.^7^, ^8^ Most recently, vibration dosimeters integrated with hand adapters^9^, ^10^ are available which enable to obtain daily exposure dose while minimizing interference with job activities.

Pneumatic tools such as rock drills, grinders, and riveters, and hammers are associated with HAVS.^11^, ^12^, ^13^, ^14^ Gasoline‐powered grass trimmers, brush cutters, leaf blowers, and chainsaws utilized in many industries including agriculture, forestry, and grounds maintenance are also significant sources of HAV, putting the workers at risk of HAVS.^15^ The U.S. Bureau of Labor Statistics (BLS) reported that there were near 1.3 million grounds maintenance workers in 2020 with a projected growth of 8% (2020–2030).^16^ Groundskeeping jobs mainly involve periodic weeding using grass trimmers along with leaf blowers but HAV exposure assessment of groundskeepers has not been performed in the United States to date.

In the present study, the HAV exposure of groundskeepers was investigated using vibration dosimeters which could account for the effects of many of the individual and work‐related factors to evaluate daily vibration exposure and tool‐specific vibration characteristics.

## 2 MATERIALS AND METHODS

The Alabama Division of Risk Management (DORM) solicited State Parks under the Alabama Department of Conservation and Natural Resources (ADCNR) to participate in this study. One State Park responded and two Park maintenance workers who regularly used power tools voluntarily participated in the study (IRB approval obtained, #IRB‐300003455).

Sampling was conducted while the participants were performing their regular groundskeeping job throughout the State Park. A total of 6 days of sampling was scheduled. All loop‐handled string grass trimmers and backpack blowers shared by the Park employees were the same gasoline‐powered model; STIHL FS 91 R for the grass trimmers and STIHL BR 430 for the backpack blowers.

Vibration evaluation including the frequency weighting was based on ISO 5349‐1:2001^1^ and measurements were conducted with palm‐strapped tri‐axial vibration dosimeters (SVANTEK SV103). The accelerometers of the dosimeters were calibrated at an acceleration of 10 m/s^2^ and frequencies of 79.58 and 159.2 Hz with a vibration calibrator (SVANTEK SV110). An accelerometer‐embedded palm adapter of the vibration dosimeters was worn on both palms of the participants.

Acceleration values from the three orthogonal axes of vibration at the 1/3 octave band center‐frequency range of 0.8–1600 Hz were collected every second throughout the sampling periods. Vibration total values, *a*_hv_, were obtained from the dosimeters and daily vibration exposure, *A*(8), was calculated using the formula below^1^:

$$A\left(8\right)=a_{\mathrm{hv}}\sqrt{\frac{T}{T_{0}}}$$

where *T* is the total daily duration of exposure to the vibration *a*_hv_ and *T*_0_ is the reference duration of 8 h.

## 3 RESULTS

The participants were right‐handed and fit/healthy with a height of 5′7″–5′9″. One participant was a 32‐year‐old White male and the other participant was a 38‐year‐old Black male. The participants had two major job duties: grounds maintenance assigned for 2–3 days and other park maintenance assigned for other 2 days with 2–3 days off for a week. Grounds maintenance job mainly involved the use of a grass trimmer and occasionally a backpack leaf blower only by participant 1. The sampling duration of each day ranged approximately 3–6 h, with a mean of 4 h 56 min. Both participants missed 1 day of sampling out of 6 sampling days scheduled.

Table 1 shows the results of *a*_hv_ and *A*(8) values for the entire sampling period. *a*_hv_ of the right and left hand of the participants ranged 3.2–8.4 m/s^2^ and corresponding *A*(8) ranged 2.2–5.4 m/s^2^. Only participant 1 was involved in operating the blower for a short duration of time on Days 1, 4, and 5. Real‐time *a*_hv_ data during those sampling days were examined to choose representative sampling periods which included both the blower and grass trimmer operations for comparison. The grass trimmer operation resulted in *a*_hv_ of 5.2–6.3 m/s^2^ for both hands, and the backpack blower operation showed *a*_hv_ of 3.3–4.6 m/s^2^ for the right hand (the only hand in contact with the blower).

**Table 1:** Vibration total values (*a*_hv_) and daily vibration exposure *A*(8) values

		Vibration total value, *a*_hv_ (m/s²)						Daily vibration exposure, *A*(8) (m/s²)					
Participant	Hand side	Day 1	Day 2	Day 3	Day4	Day 5	Day 6	Day 1	Day 2	Day 3	Day4	Day 5	Day 6
1	Right	3.6	3.5	3.9	4.1	3.5	–a	3.0	3.0	2.5	3.6	2.5	–a
	Left	3.8	3.5	3.7	4.4	–b	–a	3.2	3.1	2.4	3.8	–b	–a
2	Right	4.2	–a	7.2	5.5	3.2	4.3	3.6	–a	4.6	4.7	2.2	3.4
	Left	4.3	–a	8.4	–b	3.2	4.8	3.6	–a	5.4	–b	2.3	3.8

^a^ No sampling.

^b^ Data unavailable due to the breakage of the adapter strip during sampling.

**Figure 1:**
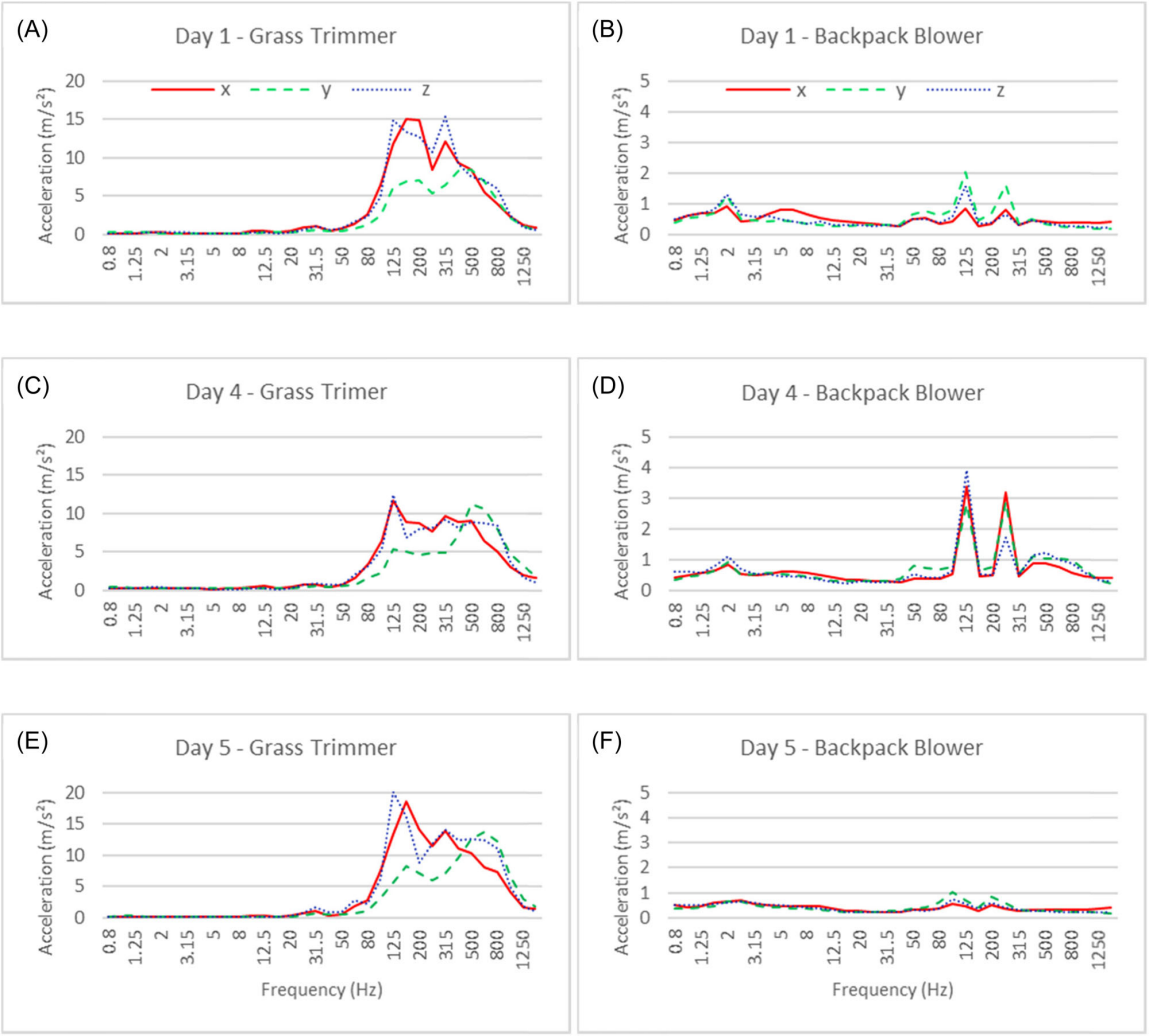
1/3 octave band frequency analysis for right‐hand grass trimmer operation (A, C, and E) and backpack blower operation (B, D, and F) of participant 1 on sampling Days 1, 4, and 5

Figure 1 shows the average right‐hand acceleration frequency spectra of both tools during the representative sampling periods. For the grass trimmer operation, two major peaks appeared at 125–160 and 315 Hz in the *x*‐ and *z*‐axes while two major peaks appeared at 125–200 and 500–630 Hz in the *y*‐axis. For the backpack blower operation, two major peaks occurred in the 100–125 and 200–250 Hz range in the three axes.

## 4 DISCUSSION

The American Conference of Governmental Industrial Hygienists (ACGIH) daily exposure limit value, 5 m/s^2^, has been reached in one sample (i.e., left hand of participant 2 on Day 3) and most samples exceeded the ACGIH action value, 2.5 m/s^2^, in either hand of both participants. However, considering the low frequency of grounds maintenance job (i.e., 2–3 times per week), the overall actual exposure throughout their employment would be much lower. Oliveira et al.^9^ used the same HAV measuring instrument as in our study to examine the HAV levels of different types of blade grass cutters during coffee mowing operations. For all equipment operating conditions, *A*(8) values exceeded 2.5 m/s^2^, and one sample from the loop‐handle grass cutter operation exceeded 5 m/s^2^.

The grass trimmer operations of participant 1 showed higher *a*_hv_ than the backpack blower operations. Compared with the literature, our grass trimmer results are lower, which would be primarily due to the different measurement methods. In Ko et al.'s^17^ study, vibration of a loop‐handle grass trimmer was measured at the handle grip using an accelerometer, which resulted in an average *a*_hv_ of 11.3 m/s^2^. Patil measured the vibration level of a bike‐handled grass trimmer to be 9.2 m/s^2^ using an accelerometer mounted near the hand grip location.^18^ A Gabasa et al.'s^8^ study which measured vibration from backpack tools at the hand‐handle interface using a hand‐held adapter obtained comparable results (1.9–3.7 m/s^2^) to our study.

High‐frequency vibration (>100 Hz) affects the hand and fingers while low‐frequency vibration (<25 Hz) is predominantly perceived in the arms and shoulder.^19^, ^20^ The grass trimmer and blower examined in this study showed their dominant frequency in the higher range (i.e., 125 Hz being the highest acceleration peak for both tools), indicating that such tools could result in higher HAV disorders in the finger/hand region.

## 5 CONCLUSION

The ACGIH daily exposure limit was reached in one sample while the overall actual exposure would be lower due to the low frequency of grounds maintenance job per week. The vibration characteristics were different between the tools and more investigations on HAV exposure are necessary to establish extensive exposure data in the United States to help mitigate the burden of HAVS.

## AUTHOR CONTRIBUTIONS

*Conceptualization, methodology, formal analysis, investigation, writing– original draft, and writing–review and editing*: Jonghwa Oh. The author has read and approved the final version of the manuscript. The corresponding author, Jonghwa Oh, had full access to all of the data in this study and takes complete responsibility for the integrity of the data and the accuracy of the data analysis.

## CONFLICT OF INTEREST

The author declares no conflict of interest.

## ETHICS STATEMENT

The study protocol (IRB‐300003455) performed at the Alabama State Park was approved by the Institutional Review Board at the University of Alabama at Birmingham and written informed consent was obtained from the participants.

## TRANSPARENCY STATEMENT

Jonghwa Oh affirms that this manuscript is an honest, accurate, and transparent account of the study being reported; that no important aspects of the study have been omitted; and that any discrepancies from the study as planned (and, if relevant, registered) have been explained.

## Data Availability

The data that support the findings of this study are available from the corresponding author upon reasonable request.
